# Genomic analysis of *Mycobacterium tuberculosis* variant *bovis* strains isolated from bovine in the state of Mato Grosso, Brazil

**DOI:** 10.3389/fvets.2022.1006090

**Published:** 2022-11-16

**Authors:** Taís Ramalho dos Anjos, Vinícius Silva Castro, Edson Silva Machado Filho, Philip Noel Suffys, Harrison Magdinier Gomes, Rafael Silva Duarte, Eduardo Eustáquio de Souza Figueiredo, Ricardo César Tavares Carvalho

**Affiliations:** ^1^Programa de Pós-graduação em Biociência Animal - Stricto Sensu, Faculdade de Medicina Veterinária, Universidade de Cuiabá (UNIC), Cuiabá, Brazil; ^2^Programa de Pós-graduação em Ciência Animal, Faculdade de Agronomia e Zootecnia, Universidade Federal de Mato Grosso (UFMT), Cuiabá, Brazil; ^3^Laboratório de Biologia Molecular Aplicado a Micobactérias, Instituto Oswaldo Cruz (IOC), Fundação Oswaldo Cruz (FIOCRUZ), Rio de Janeiro, Brazil; ^4^Laboratório de Micobactérias, Departamento de Microbiologia Médica, Instituto de Microbiologia Paulo de Góes, Universidade Federal do Rio de Janeiro (UFRJ), Rio de Janeiro, Brazil; ^5^Laboratório de Microbiologia Molecular de Alimentos, Faculdade de Nutrição, Universidade Federal de Mato Grosso (UFMT), Cuiabá, Brazil

**Keywords:** *Mycobacterium tuberculosis* variant *bovis*, mutation, phylogeny, drug resistance, zoonosis

## Abstract

The species *Mycobacterium tuberculosis* variant *bovis* (*M. tuberculosis* var. *bovis*) is associated with tuberculosis, mainly in cattle and buffaloes. This pathogen has the potential to infect other mammals, including humans. Tuberculosis caused by *M. tuberculosis* var. *bovis* is a zoonosis clinically identical to tuberculosis caused by *Mycobacterium tuberculosi*s, and the recommended treatment in humans results in the use of antibiotics. In this study, we used the whole genome sequencing (WGS) methodology Illumina NovaSeq 6000 System platform to characterize the genome of *M. tuberculosis* var. *bovis* in cattle circulating in Mato Grosso, identify mutations related to drug resistance genes, compare with other strains of *M. tuberculosis* var. *bovis* brazilian and assess potential drug resistance. Four isolates of *M. tuberculosis* var. *bovis* of cattle origin representing the main livestock circuits, which had been more prevalent in previous studies in the state of Mato Grosso, were selected for the genomic study. The genome sizes of the sequenced strains ranged from 4,306,423 to 4,332,964 bp, and the GC content was 65.6%. The four strains from Mato Grosso presented resistance genes to *pncA* (pyrazinamide), characterized as drug-resistant strains. In addition to verifying several point mutations in the *pncA, rpsA, rpsL, gid, rpoB, katG, gyrB, gyrA, tlyA, embA, embB, embC, fgd, fbiB*, and *fbiC* genes, these genes were similar to antibiotic resistance in more than 92% of the Brazilian strains. Therefore, our results indicated a high genetic diversity between our isolates and other *M. tuberculosis* var. *bovis* isolated in Brazil. Thus, multiple transmission routes of this pathogen may be present in the production chain. So, to achieve a bovine tuberculosis-free health status, the use of the WGS as a control and monitoring tool will be crucial to determine these transmission routes.

## Introduction

*Mycobacterium tuberculosis* variant *bovis (M. tuberculosis* var. *bovis*) is the etiological agent of bovine tuberculosis (bTB) (1). This pathogen belongs to the group of mycobacteria belonging to the *Mycobacterium tuberculosis* Complex (MTBC), which share genomic similarities of ~99.9% (2). *M. tuberculosis* var. *bovis* has zoonotic potential and can cause tuberculosis in humans (1). Transmission to humans can happen directly through the air, in which the pathogen is inhaled in aerosols from infected animals or carcasses, as well as indirectly through contamination through the consumption of contaminated animal products, such as raw milk and other dairy products without heat treatment, and through the consumption of undercooked or raw meat (3, 4).

This zoonosis mainly affects vulnerable people, such as HIV (human immunodeficiency virus) but also immunocompetent people (5), and is mainly related to factors such as poverty, unemployment, and a low level of education (6); it is also a public health problem and is clinically identical to tuberculosis caused by the bacterium *Mycobacterium tuberculosis* (*M. tuberculosis*), making differential diagnosis difficult (7, 8).

According to Sisco et al. (9), there is the possibility of acquiring tuberculosis through the BCG vaccine, especially in immunocompromised patients. They found a strain resistant to ethambutol, rifampicin, and isoniazid. With intrinsic or acquired antibiotic resistance, there is a need to perform drug susceptibility screening of the strain before or during patient treatment (9).

Bovine tuberculosis presents a chronic evolution characterized by the development of nodular lesions called tubercles, which can be located in any organ or tissue of the animal (10). In cases of tuberculosis in humans, treatment is carried out with the use of combinations of antibiotics, mainly rifampicin, isoniazid, ethambutol, streptomycin, ethionamide, pyrazinamide, and fluoroquinolones (6). Unlike *M. tuberculosis, M. tuberculosis* var. *bovis* is naturally resistant to pyrazinamide, one of the drugs used in the treatment of tuberculosis (11).

*M. tuberculosis* var. *bovis* have reference genes that encode proteins with known or unknown functions, such as catalase/peroxide activity (*katG* gene); encoding the DNA gyrase A subunit (g*yrA*); in the *rpoB* gene, which encodes a β subunit of RNA polymerase; RNA loops in the loops of RNA, which is encoded by the gene, related to changes in cell wall permeability (12); another example are bacteria related in genes of the *emb* region (*embA, embB*, and *embC*) with the biosynthesis of arabinogalactan and lipoarabinomannan, structural components of the tamarin wall (target ethambutol) (13).

According to Vázquez-Chacón et al. (14), there are highly reliable mutations in *M. tuberculosis* var. *bovis* that confer drug resistance. For example, mutations in the *katG* S315T genes; *rpsL* K43R or K88R; *rrs* A1401G and *gyrA* D94G confer resistance to isoniazid, streptomycin, aminoglycosides and fluoroquinolones, and these were identified in *M. tuberculosis* var. *bovis* (14). All *M. tuberculosis* var. *bovis* strains are naturally resistant to pyrazinamide (*pncA*), mutations were found at position C169G, and *Mycobacterium bovis* bacillus Calmette-Guérin (BCG) and *Mycobacterium canetti* strains are also resistant to this drug (15). The circulation of drug-resistant strains in cattle, especially those used in the treatment of tuberculosis in humans, represents a great risk for the occurrence of multidrug-resistant strains in the population (14).

Multidrug resistance (MDR) has increased worldwide, which is considered a threat to public health. Several recent investigations have reported the emergence of multidrug-resistant bacterial pathogens of different origins that increase the need for the appropriate use of antibiotics. In addition, routine application of antimicrobial susceptibility testing to detect the antibiotic of choice, as well as screening for emerging MDR strains (16–19).

The aim of this study was to characterize the genome of *M. tuberculosis* var. *bovis* in cattle circulating in Mato Grosso, identify mutations related to drug resistance genes, compare with other strains of *M. tuberculosis* var. *bovis* brazilian and assess potential drug resistance.

## Materials and methods

### Geographic area of study

**Figure 1:**
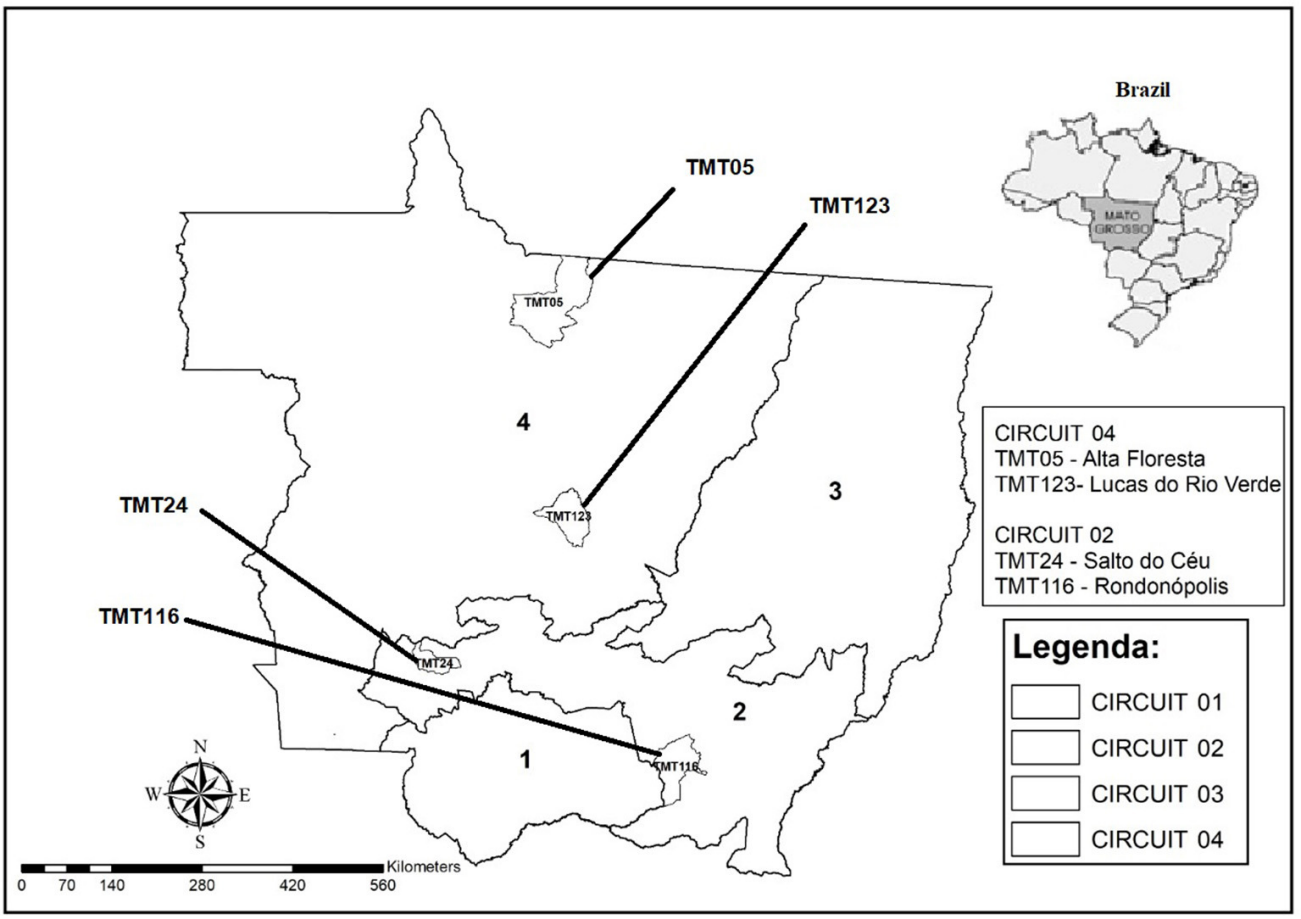
Map of Mato Grosso state with the municipalities demarcating the origins of the strains selected for genome sequencing.

This study was carried out in the state of Mato Grosso, located in midwestern Brazil (Latitude: 15° 35′ 56″ South, Longitude: 56° 5′ 42″ West), which is categorized by four livestock circuits (20), called CP 01—Pantanal; CP 02—Milk production; CP 03—Fattening and CP 04—Breeding, according to the predominant animal production characteristics, local ecosystem, and animal transit network (Figure 1).

### Sampling and zootechnical data

A total of 41 suspected bTB lesion samples of the same number of animals were collected between September 2017 and May 2018, stored in sterile packaging and frozen during the postmortem inspection procedure. The sampling was carried out in slaughterhouses in the state of Mato Grosso by Federal (SIF) and state (SISE/MT) inspection authorities using the criteria established by the regulation of inspection industrial and sanitary of animal products (RIISPOA) (21). Simultaneously, sanitary inspection and collection of suspicious lesions were carried out following normal routine at slaughterhouses in the state.

### Nucleic acid extraction from bovine tissue fragments containing suspicious lesions

Samples were divided into two aliquots, one for DNA extraction and another for culture. Sample preparation and DNA extraction from bovine tissue fragments containing suspicious lesions were performed according to Furlanetto et al. (22) using a commercial Dneasy Blood and Tissue kit (Qiagen^®^, Hilden, Germany). After extraction, the DNA was quantified by the fluorometric method using the QUBIT TM 2.0 Kit (Invitrogen^®^, Carlsbad, CA, United States).

### Nested real-time PCR (nested qPCR) of DNA extracted from animal tissue fragments

Nested q-PCR was performed using TaqMan PCR Master Mix (Applied Biosystems^®^, Foster City, CA, United States), primers and probes for the *Rv2807* gene (Applied Biosystems^®^, Foster City, CA, United States) specific for species belonging to MTBC and primers and probes for the *TbD1* region (Applied Biosystems^®^, Foster City, CA, United States) of *M. tuberculosis* var. *bovis* based on the method described by Araújo et al. (23, 24) and modified by Carvalho et al. (25).

### Bacterial isolation and identification

After detection of MTBC and *M. tuberculosis* var. *bovis* by nested q-PCR, samples positive for *Rv2807* and *TbD1* were submitted to microbiological culture, undergoing a decontamination process with 0.75% hexadecyl pyridinium chloride (HPC) and 10% sulfuric acid (H_2_SO_4_) (22, 26). After processing, the material was inoculated in duplicate in tubes with Stonebrink culture medium (Reagents: Malachite Green- C_23_H_25_CIN_2_/VETEC Rio de Janeiro, Brazil; Disodium Phosphate- NaHPO_4_/ISOFAR Rio de Janeiro, Brazil; Sodium pyruvate- C_3_H_3_NaO_3_/VETEC Rio de Janeiro, Brazil; monopotassium phosphateresiste—KH_2_PO_4_/SYNTH São Paulo, Brazil) a 2%, and incubated at 37°C under aerobic conditions for 90 days, with weekly control to record the multiplication rate, shape and recognition of characteristic colonies of mycobacteria (27–29).

The colonies suggestive of mycobacteria were the presence of bacilli identified by the technique of alcohol-acid resistant bacilli (AARB), by the Ziehl-Neelsen technique. Small, white colonies with irregular borders and a granular surface typical of *M. tuberculosis* var. *bovis* and after performing Ziehl-Neelsen, a massive presence of AARB was observed, thus, the colonies were identified as belonging to *M. tuberculosis* var. *bovis*, confirming what had been previously identified by nested q-PCR (30, 31).

### DNA extraction from *Mycobacterium tuberculosis* variant *bovis* isolates

After microbial isolation, the process of extracting DNA from the isolates was carried out by the cetyl trimethyl ammonium bromide (CTAB) enzymatic method, following the protocol described by Van Soolingen et al. (32), and quantified by a NanoDrop^®^ 2000 spectrophotometer (Thermo Scientific) to obtain a concentration of ≥ 50 ng/μL.

### Genome sequencing and data analysis

Of the 41 samples analyzed, they were submitted to nested qPCR for MTBC (*Rv2807* gene) and for *M. tuberculosis* var. *bovis* (*TbD1* gene) and were submitted to microbiological culture, through which 23 isolates with characteristic colonies of *M. tuberculosis* var. *bovis* were obtained. Of these 23 isolates, four were selected as representatives of the regions studied, adopting criteria referring to representing the main livestock circuits that had shown higher prevalence in previous studies in the state of Mato Grosso (33, 34).

Four strains were selected for genome sequencing based on each different livestock circuits in the state of Mato Grosso (20). These livestock circuits were previously established due to the higher prevalence of *M. bovis* in the state (33, 34). The state of Mato Grosso is divided into four regions, called livestock circuits (CP), according to the bovine production that is predominant in that region. Circuit 1 represents the municipalities that have a predominant production of extensive breeding; circuit 2 is representative of milk production; circuit 3 are municipalities with a fattening cycle and circuit 4 with predominant production for the rearing system (18).

Of the 41 samples analyzed, after cultivation, 23 isolates were obtained, including 17 strains of CP 2 and six strains of CP 4. The four strains were then selected for sequencing, identified as TMT24 and TMT116 (CP 2) and TMT05 and TMT123 (CP 4), which represent the municipalities of Alta Floresta (TMT05—CP4), Lucas do Rio Verde (TMT123—CP4), Salto do Céu (TMT24—CP2), and Rondonópolis (TMT116—CP2).

Sequencing was performed using the Illumina NovaSeq 6000 System platform (Illumina, Inc., San Diego, CA, USA) and S4 Flow Cell Type PE150 (Illumina, Inc., San Diego, CA, USA) and operated by GenOne Biotechnologies (Rio de Janeiro, Brazil). To assess the raw sequence quality we used a FastQC (Version 0.11.8) (35). Thereafter, we used the Trimmomatic (version 0.36) to cut Illumina adapters. The parameters used were sliding window trimming applied as operator, 4 bases to average across, and 20 bases required for average quality. To carry out the assembly, we used Shovill version 1.1.0 (https://github.com/tseemann/shovill), with Spades used as an assembler, a depth of 100, a minimum contig length of 200 bp and a minimum coverage of 5 (36). After the generation of contigs, genomic annotation was obtained using Prokka version 1.14.5, with default parameters applied (37), and the presence of resistance and virulence genes was analyzed *in silico*. Nevertheless, other specific analyses for TB, such as the determination of the strain's lineage, spoligotyping *in silico*, and detection of resistance genes and mutations, we used TB profiler version 2.8.14 (https://github.com/jodyphelan/TBProfiler) (38). To check the assembly sequencing quality we used Quast (version 5.0.2) (39) with default parameters applied.

### Data collection and construction of the phylogenetic tree

**Figure 2:**
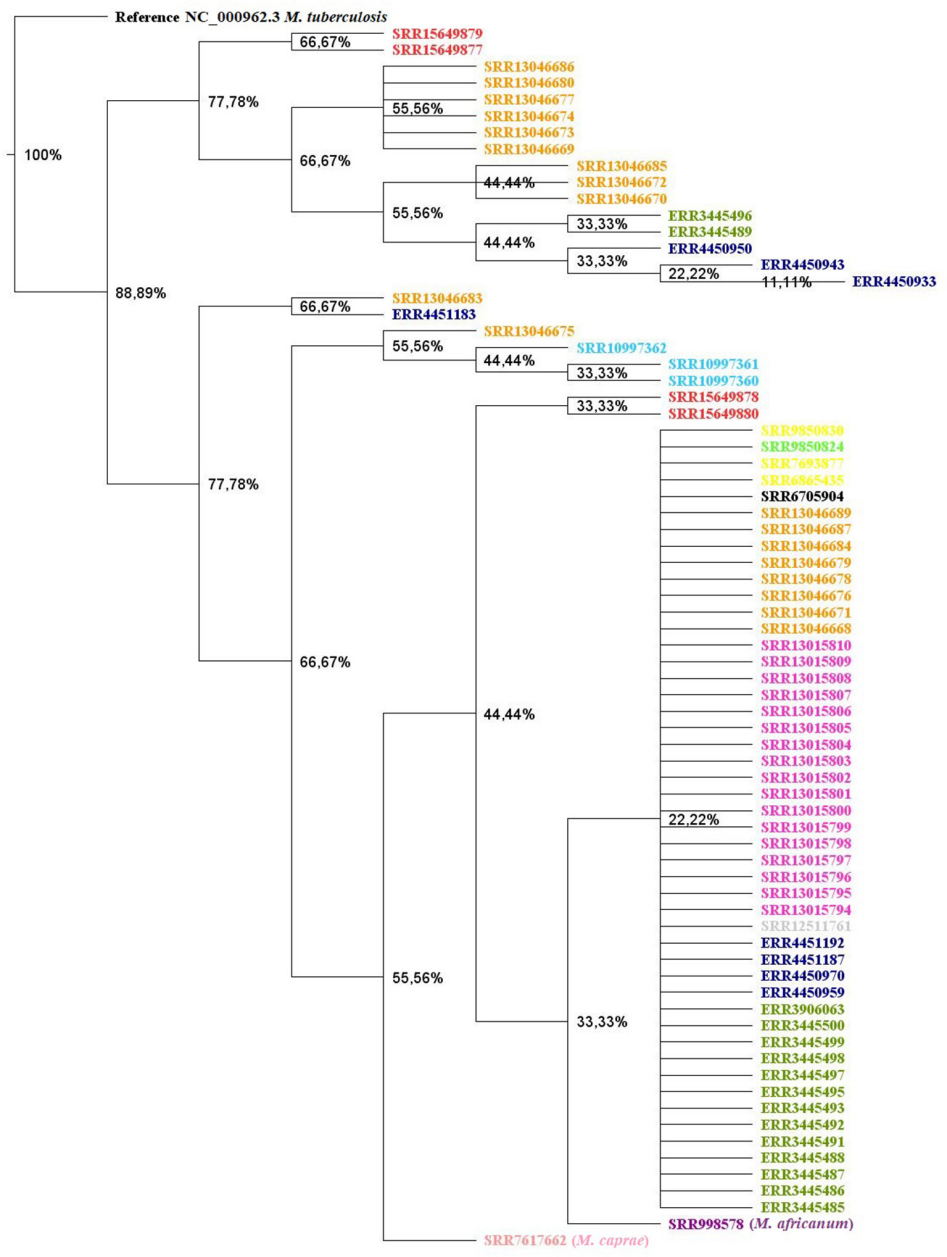
Maximum likelihood phylogenetic tree of *Mycobacterium tuberculosis* variant *bovis* genomes sequenced in the state of Mato Grosso grouped with other genomes sequenced in other states of Brazil. Four *Mycobacterium tuberculosis* variant *bovis* genomes isolated from bovine TMT05 (SRR15649880), TMT116 (SRR15649878), TMT24 (SRR15649879), and TMT123 (SRR15649877) from the state of Mato Grosso. Identifications in **red**: *Mycobacterium tuberculosis* variant *bovis* genomes obtained from cattle and sequenced in this study; **green**: *Mycobacterium tuberculosis* variant *bovis* genomes from cattle or buffalo from Marajó Island, Pará; **orange**: genomes of *Mycobacterium tuberculosis* variant *bovis* obtained from cattle or buffaloes from the Amazon; **dark blue**: *Mycobacterium tuberculosis* variant *bovis* genomes obtained from cattle from southern Brazil; **pink**: *Mycobacterium tuberculosis* variant *bovis* genomes obtained from deer from a safari in southern Brazil; **yellow**: llama; **light blue**: BCG strain genome; **light green**: capybara; **gray**: *Mycobacterium tuberculosis* variant *bovis* isolated from cattle; black: first *Mycobacterium tuberculosis* variant *bovis* strain sequenced in Brazil; outgroups: *Mycobacterium africanum* **(purple)**, *Mycobacterium caprae* **(light pink)**, and *Mycobacterium tuberculosis* **(black)**.

A total of 76 *M. tuberculosis* var. *bovis* genomes were used to construct the phylogenetic tree. Of these 76 genomes, four are the genomes obtained in the present study, another 49 SRA were obtained from the NCBI (Keywords: *Mycobacterium bovis* AND Brazil), and another 23 sequences were identified in scientific articles published but not covered by the NCBI Search (Supplementary material 1). Among the sequences, we selected the first *M. tuberculosis* var. *bovis* strain sequenced in Brazil, isolated from a bovine in 2010 in the state of São Paulo, of identification SRA: SRR6705904 (40). However, in a recent study carried out by Rodrigues et al. (33), 74 *M. tuberculosis* var. *bovis* genomes were made available at NCBI under BioProject PRJEB39667. However, as a convenience, we divided the 74 samples into eight different clades, as specified in the authors' phylogenetic tree, and analyzed one strain belonging to each clade (41). Additionally, 19 sequences were included belonging to the study performed by Conceição et al. (42) in a study of *M. tuberculosis* var. *bovis* isolated from Ilha de Marajó (42). A total of 19 genomes came from a study on *M. tuberculosis* var. *bovis* in animals raised in Amazonas under BioProject PRJNA675550. Another 17 genomes were derived from *M. tuberculosis* var. *bovis* isolated in deer in a safari park in southern Brazil under the BioProject PRJNA675476 (35). Finally, other genomes isolated from wild animals were included, such as 3 genomes from the isolation of *M. tuberculosis* var. *bovis* in llamas (SRR6865435, SRR7693877, SRR9850830) and 1 genome in capybara (SRR9850824). Another 3 strains described as BCG (Bacillus Calmette Guerin) were also inserted into the phylogenetic tree (9). Finally, 1 single genome (SRR12511761) from a bovine isolate was inserted into the construction of the phylogenetic tree. We added three outgroups, *M. africanum, M. caprae*, and *M. tuberculosis*, for better visualization of the clades. To construct the phylogenetic tree, the data were submitted to the Snippy package version 4.6.0 (https://github.com/tseemann/snippy). This tool was used to perform variant calling and core-genome alignment. For this, paired-end reads of all strains were analyzed using a minimum mapping quality of 60, with a minimum coverage of 10x, and a minimum proportion for variant evidence of 0.9. *Mycobacterium tuberculosis* H37Rv (NC_000962.3) was used as a reference strain for creating the core alignment. Subsequently, all multiple Snippy outputs from the core alignment with variant calling were combined into a SNP core alignment using Snippy-core version 4.6.0 (https://github.com/tseemann/snippy). Thus, the core-genome SNP alignment was used to perform a phylogenetic tree using the approximately maximum likelihood phylogenetic trees method with FastTree v. 2.1.11, with a site rate of 20, and turn off the minimum-evolution nearest-neighbor interchanges (NNIs) and minimum-evolution subtree-pruning-regrafting (SPRs). The phylogenetic tree image was obtained using Geneious Prime (v2021.2.2) and annotated using FigTree v1.4.4 (Figure 2) (39). An important point was that we removed four strains (ERR3445501, ERR3445502, ERR3445503, and ERR3445504) after generating the phylogenetic tree because possible DNA contamination may occur, one time that RDscan and the phylogenetic tree showed an unusual pattern.

### Identification of regions of difference in Brazilian *Mycobacterium tuberculosis* variant *bovis* strains

RDscan (https://github.com/dbespiatykh/RDscan) was used to verify the deletion regions (RDs) in the analyzed genomes to understand the differentiation of *M. tuberculosis* var. *bovis* strains from Mato Grosso with the reference strain (Mbovis_AF212297) and the other Brazilian strains.

## Results

### Nested qPCR and microbiological culture

From the 41 suspected bTB lesions collected according to Brazilian official judgment criteria, 100% (41/41) positive for MTBC, 70.7% (29/41) positive for *M. tuberculosis* var. *bovis* in nested qPCR were submitted to cultivation, and 56% (23/41) generated colonies suggestive of *M. tuberculosis* var. *bovis* in Stonebrink medium. All were submitted and confirmed by this q-PCR, and four strains were selected for sequencing to represent two livestock circuits in the state of Mato Grosso, not only within a single circuit, in addition to selecting strains of *M. tuberculosis* var. *bovis*, which in previous studies were more prevalent (33, 34).

### Sequencing, assembly, and genome annotation of the sequenced *M. tuberculosis* var. *bovis* strains

*M. tuberculosis* var. *bovis* genomic DNA was sequenced on an Illumina NovaSeq 6000 System platform with 28x to 31x coverage. DNA sequencing and assembly generated 4,306,423 to 4,332,964 nucleotides and a GC content of 65.6. As a result of the genome reading, the number of contig values obtained ranged from 70 to 95. With the Quast version 5.0.2 tools, we obtained N50 values that ranged from 137.022 to 205.012. Table 1 describes the size of the genomes, the number of CDSs that varied from 3,981 to 4,010, and other assembly and annotation results.

**Table 1:** Assembly and annotation of sequenced *Mycobacterium tuberculosis* variant *bovis* genomes.

**Strains**	**TMT05**	**TMT24**	**TMT116**	**TMT123**
Total of reads	505,512	340,469	296,049	269,963
No. of contigs	70	94	76	95
N50 (bp)	205,012	16,519	137,022	137,022
Genome size (bp)	4,327,320	4,332,203	4,306,423	4,332,964
No. of CDSs	3,997	401	3,981	3,999
rRNA	3	3	3	3
Repeat region	2	2	2	2
tRNA	52	52	52	52
tmRNA	1	1	1	1
Sequencing depth (x)	28	31	28	30
G+C contents (%)	65.61	65.62	65.62	65.62

pb, base pairs; CDS, coding sequence; rRNA, ribosomal ribonucleic acid; tRNA, transfer ribonucleic acid; tmRNA, transfer messenger ribonucleic acid.

### Lineage, resistance genes, and mutations found in *Mycobacterium tuberculosis* variant *bovis* strains

To characterize the strains to the lineage level, spoligotyping *in silico*, and verification of the presence of resistance-associated genes, we used TB profiler version 2.8.14 software. Our results show that all strains in this study TMT05 (SRR15649880), TMT116 sample (SRR15649878), TMT24 (SRR15649879), and TMT123 (SRR15649877) belonged to the *M. tuberculosis* var. *bovis* lineage BOV AFRI and the spoligotype BOV 1; BOV 2. Another 71 genomes belong to the BOV 1 spoligotype, BOV 11, with the exception of two genomes (ERR3445502, ERR3445504).

The *pncA* gene, which is responsible for pyrazinamide resistance, was detected in all sequenced strains in this study, as expected. As in the other 71 Brazilian strains, with the exception of the two strains (ERR3445502, ERR3445504). Using Prokka version 1.14.5, it was possible to detect other genes that are associated with resistance to several antimicrobials, *pncA* and *rpsA* (pyrazinamide); *katG, inhA, ahpC* and *glf* (isoniazid); *rpoB* (rifampicin); *rpsL* and *gid* (streptomycin); *embB, embB* and *embC* (ethambutol); *inhA, ethA* and *ethR* (ethionamide); *gyrA* and *gyrB* (florquinolones); *eis* (kanamycin/amikacin); *tlyA* (capreomycin); *thyA* and *ribD* (paraminosalicylic acid); *alr, ddl* and *cycA* (cycloserine); *atpE* and *mmpR5* (bedaquiline); *rplC* (linezolid); *ddn, fgd1, fbiA, fbiB, fbiC*, and *fbiD* (delamanid) (6, 43–48), according to Table 2.

**Table 2:** Resistance genes in *Mycobacterium tuberculosis* variant *bovis* strains isolated in Mato Grosso associated with antibiotics routinely used in the treatment of human TB.

**Drug or drug class**	**Resistance genes**
Pyrazinamide	*pncA, rpsA*
Isoniazid	*katG, inhA, ahpC*, and *glf*
Rifampicin	*rpoB*
Streptomycin	*rpsL, gid*
Ethambutol	*embA, embB*, and *embC*
Ethionamide	*inhA, ethA*, and *ethR*
Quinolones	*gyrA* and *gyrB*
Kanamycin/amikacin	*Eis*
Capreomycin	*tlyA*
Paraminosalicylic Acid	*thyA* and *ribD*
Cycloserine	*alr, ddl*, and *cycA*
Bedaquiline	*atpE* and *mmpR5*
Linezolid	*rplC*
Delamanid	*ddn, fgd1, fbiA, fbiB, fbiC*, and *fbiD*

Mutations were detected in the genes *pncA, rpsA, rpsL, gid, rpoB, katG, gyrB, gyrA, tlyA, embA, embB, embC, fgd1, fbiB*, and *fbiC*, as described in Table 3 and Supplementary material 2.

**Table 3:** Mutations present in *Mycobacterium tuberculosis* variant *bovis* strains isolated from caseous lesions obtained from bovine carcasses in the state of Mato Grosso, Brazil, with other Brazilian strains.

**Gene**	**Position**	**Mutation**	**Mato Grosso strains** **frequency (*n* = 4)**	**Brazilian strains** **(*n* = 77)**	**Mutations in** **Brazilian** **strains (%)**
*pnc*A	57	His Asp	4	75	97.4
*rpsA*	440	Ala Thr	4	73	94.8
*rpsL*	165	T>C	4	75	97.4
*Gid*	615	T>C	4	73	94.8
*rpoB*	3,225	T>C	4	73	94.8
*katG*	463	Arg Leu	4	74	96.1
*katG*	609	G>A	4	74	96.1
*katG*	87	G>T	4	74	96.1
*gyrB*	513	G>A	4	73	94.8
*gyrB*	1,167	C>T	4	74	96.1
*gyrB*	403	Ala Ser	4	74	96.1
*gyrA*	21	Glu Gln	4	77	100
*gyrA*	95	Ser Thr	4	77	100
*gyrA*	984	C>T	4	73	94.8
*gyrA*	1,842	T>C	4	76	98.7
*gyrA*	639	Asp Ala	4	71	92.2
*gyrA*	668	Gly Asp	4	75	97.4
*tlyA*	33	A>G	4	71	92.2
*embA*	988	C>T	4	75	97.4
*embB*	13	Asn Ser	4	74	96.1
*embB*	351	C>T	4	74	96.1
*embB*	378	Glu Ala	4	74	96.1
*embC*	270	Thr Ile	4	74	96.1
*embC*	2,781	C>T	4	74	96.1
*embC*	3,108	C>T	4	74	96.1
*fgd1*	960	T>C	4	73	94.8
*fbiB*	315	Asp Ala	3^*^	42	54.5
*fbiC*	32	A>G	4	75	97.4

* Except TMT05 strain.

The strains sequenced in this work SRR15649878 (TMT116), SRR15649879 (TMT24), SRR15649877 (TMT123), and SRR15649880 (TMT05) as well as 68 Brazilian *M. tuberculosis* var. *bovis* strains were classified as drug resistant using the TBProfiler. Another three BCG strains (SRR10997360, SRR10997362, and SRR10997361) were classified as multidrug resistant (MDR), and another strain, ERR3445487, was also classified as MDR (Table 4).

**Table 4:** Drug-resistant TBProfiler report.

**Strains**	**Host**	**Genome position**	**Locus**	**Tag gene**	**Change**	**Estimated fraction**	**Drug**	**Drug-resistance** **(TBprofiler)**
SRR15649878 (TMT116)	Bovine	2289073	Rv2043c	*pncA*	His57Asp	1.000	Pyrazinamide	Drug-resistant
SRR15649879 (TMT24)	Bovine	2289073	Rv2043c	*pncA*	His57Asp	1.000	Pyrazinamide	Drug-resistant
SRR15649877 (TMT123)	Bovine	2289073	Rv2043c	*pncA*	His57Asp	1.000	Pyrazinamide	Drug-resistant
SRR15649880 (TMT05)	Bovine	2289073	Rv2043c	*pncA*	His57Asp	1.000	Pyrazinamide	Drug-resistant
SRR10997360	BCG Culture	761110	Rv0667	*rpoB*	Asp435Val	1.000	Rifampicin	MDR
		2155168	Rv1908c	*katG*	Ser315Thr	1.000	Isoniazid	
		2289073	Rv2043c	*pncA*	His57Asp	1.000	Pyrazinamide	
SRR10997362	BCG Culture	761161	Rv0667	*rpoB*	Leu452Pro	0.993	Rifampicin	Pre-MDR
		2289073	Rv2043c	*pncA*	His57Asp	0.985	Pyrazinamide	
SRR10997361	BCG Culture	761110	Rv0667	*rpoB*	Asp435Val	1.000	Rifampicin	MDR
		2155168	Rv1908c	*katG*	Ser315Thr	1.000	Isoniazid	
		2289073	Rv2043c	*pncA*	His57Asp	1.000	Pyrazinamide	
ERR3445487	Buffalo or	1472644	rrs	*rrs*	799c>t	0.683	Streptomycin	Other
	Bovine	2289073	Rv2043c	*pncA*	His57Asp	1.000	Pyrazinamide	
All other 69 genomes	-	2289073	Rv2043c	*pncA*	His57Asp	-	Pyrazinamide	Other

### Genomic analysis of *M. tuberculosis* var. *bovis* strains

Illumina raw data [sequence read archive (SRA)] and the contigs genome with respective annotations were deposited with the NCBI and are described in Table 4. All SRA and genome annotation data were included under BioProject number PRJNA756983 (Table 5).

**Table 5:** Data on strains deposited at the NCBI.

**Strains**	**Lineage**	**Spoligotype**	**BioSample** **accession no**.	**SRA accession no**.	**Assembly accession no**.
TMT05	*M. bovis* BOV AFRI	BOV 1; BOV 2	SAMN21018175	SRR15649880	JAIOHE000000000
TMT24	*M. bovis* BOV AFRI	BOV 1; BOV 2	SAMN21018176	SRR15649879	JAIOHF000000000
TMT116	*M. bovis* BOV AFRI	BOV 1; BOV 2	SAMN21018177	SRR15649878	JAIOHG000000000
TMT123	*M. bovis* BOV AFRI	BOV 1; BOV 2	SAMN21018178	SRR15649877	JAIOHH000000000

In the phylogenetic tree (Figure 2), two large branches were determined, with a bootstrap value of 77.7% for each branch, called a clade. Through the phylogenetic tree, it can be observed that the lineages of *M. tuberculosis* var. *bovis* from the state of Mato Grosso are distributed in both clades.

Deletion regions (RDs) were identified in the four strains sequenced in this study as well as in the other 71 genomes analyzed, except for two genomes (ERR3445502; ERR3445503), through RDscan, including RD4, RD5, RD6, RD7, RD8, RD9, RD10, RD12, and RD13 (Supplementary material 4).

Some regions were deleted (*Rv0095c, Rv0867c*, and *Rv1563c*), and others were preserved (*Rv3798*) in the four strains sequenced in this study compared to the reference strain (Mbovis_AF212297). The Mato Grosso strain SRR15649880 (TMT05) showed the absence of the RDoryx_1 region and the *Rv3508* gene, which differs from the reference strain.

The SRR15649878 (TMT116) genome showed deletion of the *Rv0578c, RD145, Rv3511, Rv3512, Rv3513c*, and *Rv3514* regions and preservation of the RDoryx_4, RD182, *Rv1361c, Rv1758, Rv1757c, Rv1756c, Rv2168c*, and *Rv2167c* regions of the reference.

## Discussion

The *M. tuberculosis* var. *bovis* lineage was identified by *in silico* spoligotyping in the four strains sequenced in this work TMT05 (SRR15649880), sample TMT116 (SRR15649878), TMT24 (SRR15649879), and TMT123 (SRR15649877) and belonged to the lineage BOV AFRI and BOV1 BOV2, the same lineage identified in another study of *M. tuberculosis* var. *bovis* in cattle from Bahia, Brazil (49). Another 71 genomes belong to the BOV1 spoligotype, BOV11, with the exception of two genomes (ERR3445502, ERR3445504).

According to Smith (50), the African 1 and African 2 clonal complexes are never or rarely found in cattle outside Africa. Carneiro et al. (51) pointed out that the current clonal complexes may not represent all the diversity of the *M. tuberculosis* var. *bovis* lineage and identified for the first time in Brazil the Lb1 lineage, and some strains identified in this lineage carry the Af2 clonal complex marker, frequently found in East Africa, demonstrating the importance of connections of this lineage with the African continent (51).

Due to Africa's proximity to countries such as Portugal and its colonies, hypothetically, strains may have been introduced for the first time in the Amazon region during the colonization of Brazil (51) and have been dispersed to other states in the country.

In addition to the presence of *M. tuberculosis* var. *bovis*, other important point is the emergence of drug resistance as serious threat to the control of human tuberculosis, as strains that are resistant to multiple drugs severely limit treatment options (52). With the results obtained in this work, it is worrisome because strains have the potential for resistance to many antibiotics, and there are few treatment options left in cases of zoonotic tuberculosis.

According to Mota et al. (53), there are 16 antibiotics known to have an effective action on any bacillus capable of causing tuberculosis in humans, and of these, six are of preferential use in the treatment of tuberculosis in humans, namely, streptomycin, rifampicin, ethambutol, pyrazinamide, ethionamide and isoniazid.

The main genes detected were *pncA, katG, inhA, ahpC, glf, rpoB, rpsL, embA, embB, embC, inhA, ethA, ethR, gyrA, gyrB, eis, tlyA, thyA, ribD, alr, ddl, cycA, atpE, mmpR5, rplC, ddn, fgd1, fbiA, fbiB, fbiC*, and *fbiD*, according to Table 2. We detected point mutations in the genes *pncA, rpsA, rpsL, gid, rpoB, katG, gyrB, gyrA, tlyA, embA, embB, embC, fgd1, fbiB*, and *fbiC*, as described in Table 3. A limitation of the study is that the phenotypic analysis for comparison with the genotypic results has not yet been possible, but it is a future project to be done.

To improve successful treatment outcomes in cases of tuberculosis caused by multidrug resistant strains, 68 countries started using the drug bedaquiline, and 42 countries started using the drug delamanid at year-end 2017 (54); however, both drugs are not available in Brazil (6), and the strains sequenced presently demonstrated the presence of *atpE* and *ddn* genes that can confer resistance to these two drugs, respectively.

Multidrug-resistant strains of *M. tuberculosis* var. *bovis* hamper TB control and exhaust treatment options in humans (55–57). Gómez-Gonzales et al. (58) found variants in genes that can confer resistance to bedaquiline and delamanid in more than 33,000 *M. tuberculosis* isolates collected from humans even before the launch of these new drugs, suggesting an intrinsic resistance of the strains. This fact constitutes new obstacles that threaten the global control of TB (58).

Acquired resistance to anti-TB drugs occurs due to spontaneous mutations, including single nucleotide polymorphisms (SNPs) and insertions and deletions (indels), in genes encoding drug targets (58). Antibiotic resistance can occur spontaneously, even without antimicrobial exposure. As a result, drug sensitivity tests, including rapid molecular techniques, are needed for the accurate diagnosis and treatment of TB (59).

Vázquez-Chacón et al. (14) identified drug resistance mutations among *M. tuberculosis* var. *bovis* strains in the Americas. Their results showed that *M. tuberculosis* var. *bovis* isolates from animal strains harbor mutations that confer resistance to first- and second-line antibiotics, resistance to isoniazid, fluoroquinolones, streptomycin and aminoglycosides. These results highlight the importance of molecular surveillance to monitor the emergence of mutations that are associated with multidrug drug resistance in cattle and other non-human mammals (14). However, they did not find mutations in genes related to next-generation drugs such as delamid and bedaquiline.

According to Miotto et al. (60), there are high confidence mutations that confer antibiotic resistance *in M. tuberculosis*. The frequency of mutations in resistant and susceptible strains was calculated using statistical measures to classify mutations as high, moderate, minimal or indeterminate confidence to predict resistance. They found resistance-associated mutations for rifampicin, isoniazid, ofloxacin/levofloxacin, moxifloxacin, amikacin, kanamycin, capreomycin, streptomycin, ethionamide/prothionamide, and pyrazinamide (60). With the mutations identified in the *M. tuberculosis* var. *bovis* strains sequenced in this work, only the *gyrA* gene was associated with the results of Miotto et al. (60), in which one of the mutations in the *gyrA* gene was associated with not being related to resistance to the antibiotic moxifloxacin; that is, this mutation does not interfere with resistance, but the other mutations could not be associated.

The most common target genes associated with streptomycin resistance are *rrs, rpsL*, and *gidB* (11). The *rpsL* and *gid* genes were detected, and mutations were found in all isolates sequenced in this study as well as in most Brazilian strains of *M. tuberculosis* var. *bovis*. Studies carried out by Djeman et al. (11) also detected a streptomycin-resistant *M. tuberculosis* var. *bovis* isolate; according to the authors, their study was the first to describe a streptomycin-resistant *M. tuberculosis* var. *bovis* isolate of animal origin. Strains capable of causing multidrug resistance tuberculosis are a threat to human medicine, as they cause treatment failures and, in cases where it is not possible to treat the infection, lead to the death of the patient.

With studies in which good results were obtained, that is, cure of treated animals (53, 61), on the action of isoniazid in the treatment of bTB intermittently, using high doses for prolonged periods, the use of this drug in the treatment of bTB in herds infected with *M. tuberculosis* var. *bovis* has become routine, mainly in the dairy basins of Minas Gerais and São Paulo. The use of isoniazid in the routine treatment of bTB has become common, and milk cooperatives make this drug available in large quantities without any control, characterizing its indiscriminate use (53). This fact may be one of the reasons why *M. tuberculosis* var. *bovis* becomes resistant to isoniazid.

Antibiotics from the quinolone group are increasingly used in the treatment of respiratory infectious diseases in humans, which has led the strains of *M. tuberculosis* to be resistant to this drug (62). Mutations in the *gyrA* and *gyrB* genes are associated with quinolone resistance in *M. tuberculosis*, and these residues are thought to play a role in drug binding and quinolone resistance (62). Mutations in *gyrA* confer high-level resistance, while mutations in *gyrB* confer low-level resistance (52, 63).

In addition, there is an aggravating factor, and cases of patients with *M. tuberculosis* var. *bovis* and *M. tuberculosis* coinfection have been reported in urban areas (64). National studies have been restricted to urban areas, but rural areas should be investigated, since the occupational character of the disease mainly affects individuals who deal directly with live animals, handlers, breeders, veterinarians, and workers in meat, dairy and laboratories (6).

The strains currently show the same patterns in the detection of genes and mutations that can confer resistance to antibiotics; they are part of the same geographic area, which is the state of Mato Grosso, but they belong to very different livestock circuits. We also verified that the same conditions in more than 92% of the Brazilian strains of *M. tuberculosis* var. *bovis* presented similar patterns to those of Mato Grosso.

With the phylogenetic tree, we can verify two clades using a bootstrap above 77.7% and that the genomes sequenced in this work are distributed in both clades. The genomes ERR3445501, ERR3445502, ERR3445503, ERR3445504 were external to the clades, with ERR3445502 and ERR3445504 probably belonging to another species of the MTC complex that was inserted in the NCBI as *M. tuberculosis* var. *bovis*, so we removed these four genomes when generating the phylogenetic tree because they distort the branches of the tree. The explanation of these four genomes being external to the clades can be explained by the results of the RDscan, which did not identify the deletion regions preserved in *M. tuberculosis* var. *bovis* (RD4-RD10; RD12; RD13) in the ERR3445502 and ERR3445504 genomes and in the ERR3445503 genome RD9 deletion region identified (Supplementary material 4). The strains external to the clades ERR3445502, ERR3445503 and ERR3445504 were classified as MRD or pre-MDR and showed resistance to the drugs ciprofloxacin, fluoroquinolones, levofloxacin, moxifloxacin, ofloxacin (mutation in *gyrB*) and streptomycin (mutation in *rrs*), with the strains ERR3445504; pyrazinamide (*pncA*), isoniazid (*kasA*), and ethambutol (*embB*) strains ERR3445501 and ERR3445503; and isoniazid (*kasA*) strain (ERR3445502; Supplementary material 3).

The three BCG strains (SRR10997360, SRR10997362, and SRR10997361) were grouped together in a branch and were classified as MDR (Table 4). In addition to being resistant to pyrazinamide, they were resistant to other drugs. The BCG strains (SRR10997360 and SRR10997361) showed mutations in the *rpoB* (rifampicin), *katG* (isoniazid) and *pncA* (pyrazinamide) genes; the BCG strain (SRR10997362) showed mutations in *rpoB* and *pncA*; strain SRR13046675 showed mutation only in *pncA*. The other Brazilian strains showed only drug resistance to *pncA*. These results were reported by TB profiler, who compares the results of mutations already recognized in the literature with the confirmation that that particular mutation is truly linked to drug resistance. Strain ERR3445487 showed mutations in *rrs* (streptomycin) and *pncA*, also classified as MDR.

Although we selected four genomes from four different municipalities in the state of Mato Grosso, coming from different properties, a limitation of our study is the small sample size, which makes it difficult to interpret the reality of bTB in the state. A small number of animal carcasses with lesions suggestive of bTB were included in the genomic study. However, they are the first lineages sequenced in the state of Mato Grosso and, therefore, the first data on the genomics of *M. tuberculosis* var. *bovis* in this region, with the potential to help the epidemiological surveillance system in the state.

Regarding livestock circuits (CP), the genomes did not remain in the same clade, and there was no association of livestock circuits in this sample, with samples TMT05 and TMT123 representing the livestock circuit (CP4) and the livestock circuits TMT24 and TMT116.

In this study, we provide the first sequencing-based description of the population structure of *M. tuberculosis* var. *bovis* in the state of Mato Grosso, central-west region of Brazil. Other studies in the future may sequence a greater number of isolates from more herds, focusing on different livestock circuits and relating them to genetic diversity within the state, with the obtainment of available bovine movement data.

According to Carneiro et al. (51), in the Amazonas region, no significant difference was observed in the distribution of genetic diversity between the hosts (bovine and buffalo). In the study carried out by Conceição et al. (42) from Ilha de Marajó, it was not possible to verify these data because the information on the hosts is not available in the database.

Because the genomes of Ilha de Marajó are strongly related and form part of a clade and were probably introduced in the region during a single event still unknown (51), in contrast, the genomes of the Amazonas region were determined in three different events, not knowing the order of these events, which may have originated from neighboring cities, originated from Ilha de Marajó and even imported from other states (51). There are still many answers to be answered regarding the true epidemiological picture of bTB both in the state of Mato Grosso and other states in Brazil. It is necessary to know the genetic profile and understand the transmission routes of *M. tuberculosis* var. *bovis*, as it is essential to assist in the control and eradication of bTB.

The genetic profile of the genomes generated in this study should be further explored, including verification of possible specific genetic regions to strains from Mato Grosso, developing specific primers and probes to be used in the epidemiological investigation of the region.

Additionally, cultural aspects, such as consuming products derived from unpasteurized raw milk and consuming raw or undercooked meat, can pose a threat to human health (65). Mato Grosso is a large state and is strongly linked with livestock, and many rural workers have contact with animals and can be infected with *M. tuberculosis* var. *bovis*.

It needs to be investigated whether this zoonotic disease underestimates the risk of bTB for humans in the state of Mato Grosso. We believe that the information generated in this work is essential and that it can contribute to strategies for the control and eradication of bTB in the center-west of Brazil, reducing the risk to human health and ensuring food safety. These results indicate that epidemiological surveillance in Mato Grosso should invest in controlling import of cattle and/or buffaloes from neighboring states, such as Pará and Amazonas, which tested negative for bTB. Additionally, bTB control programs should invest in encouraging disease control, especially in the milk production region, which is represented by livestock circuit 2 (CP2), where there is a greater number of bTB outbreaks. In addition to having stricter public policies regarding the indiscriminate use of antimicrobials used in cattle, it can cause resistance to drugs used in the treatment of TB in humans.

The four strains sequenced in this study as well as 71 other genomes analyzed, except for two genomes (ERR3445502; ERR3445503), showed the deleted regions RD4, RD5, RD6, RD7, RD8, RD9, RD10, RD12, and RD13, which are highly conserved in *M. tuberculosis* var. *bovis* (30).

The genome that observed more deletion regions of Mato Grosso strains was SRR15649878 (TMT116). Three regions with deletions (RD) (Table 4), different from the reference strain, in the TMT116 strain in the milk region in the state of Mato Grosso, meaning a strain that had a greater number of mutations and more evolved in a region that has a greater number of bovine tuberculosis cases. The Mato Grosso strain SRR15649880 (TMT05) showed the absence of the *Rv3508* gene that may be correlated with the response to the inhibition of aerobic respiration in mycobacteria (66).

The *Rv0095c* gene is present in the reference strain but deleted in the four strains sequenced in this study as well as in most Brazilian strains, except (SRR12511761; SRR9850830). The *Rv0095c* gene, a conserved protein of unknown function, has been associated with the successful transmission of a clade of *M. tuberculosis* in Peru (67).

The *Rv0867c* gene was not deleted in the four strains sequenced in this work or in another 60 Brazilian strains, different from the reference strain. This gene represents one of five *Rpf* (secreted growth factor)-like genes that are expressed in actively growing cells, stimulate bacterial growth at low concentrations and are related to dormant cell resuscitation (68). The four strains sequenced in this work do not have the *Rv1563c* gene deletion, as well as the other 72 Brazilian strains, which were deleted in the reference strain.

The *Rv3798* gene is deleted in the four sequenced strains and in another 59 Brazilian strains and is preserved in the reference strain. It was not possible to identify a distinct regional genomic characteristic in the Mato Grosso strains compared to other Brazilian strains.

Through genome sequencing, it was possible to characterize *M. tuberculosis* var. *bovis* in samples of caseous lesions suggestive of bovine tuberculosis obtained during slaughter in slaughterhouses in the state of Mato Grosso, Brazil. The size of the genomes ranged from 4,306,423 to 4,332,964 bp; there was a number of CDs that ranged from 3,981 to 4,010 and identified that the sequenced strains of *M. tuberculosis* var. *bovis* are part of the BOV-AFRI strain. Comparing the *M. tuberculosis* var. *bovis* strains sequenced in this work with other strains sequenced in Brazil, it was possible to verify that the genomes were divided into the two clades (bootstrap above 77.7%) and that they are in different branches within these clades. The *M. tuberculosis* var. *bovis* strains from Mato Grosso sequenced in this work were determined to be drug-resistant strains. Future phenotypic analyses are needed to determine their real potential for resistance to other drugs, in addition to pyrazinamide resistance, as other mutations (*rpsA, rpsL, gid, rpoB, katG, gyrB, gyrA, tlyA, embA, embB, embC, fgd, fbiB*, and *fbiC*) were found in genes responsible for resistance to several antibiotics used in the treatment of tuberculosis in humans, similar to most brazilian *M. tuberculosis* var. *bovis* strains.

With these results, being the first sequencing of the genome of *M. tuberculosis* variant *bovis* in the State of Mato Grosso, we believe that these results are essential for the control and eradication of bTB in the State, reducing the risk to human health and ensuring food security. We emphasize the need to expand the use of the WGS in the Bovine Tuberculosis Control and Eradication Programs, with the objective of effectively achieving the eradication of the disease, not only in the State of Mato Grosso, which is the largest producer of cattle in the country, but also in other countries. brazilian states, in order to obtain bovine tuberculosis-free health status.

## Data availability statement

The datasets presented in this study can be found in online repositories. The names of the repository/repositories and accession number(s) can be found in the article/Supplementary material.

## Ethics statement

The animal study was reviewed and approved by Comissão de Ética no Uso de Animais da Universidade de Cuiabá (CEUA/UNIC). Written informed consent for participation was not obtained from the owners because notifiable zoonosis diagnosis.

## Author contributions

Material preparations were performed by TA and RC. Data analysis was performed by TA, VC, EF, RC, EM, and PS. The first manuscript was written by TA. All authors contributed to the study conception and design, corrected the previous versions of the manuscript, read, and approved the final manuscript text.

## Funding

This study was financed in part by the Coordenação de Aperfeiçoamento de Pessoal de Nível Superior—Brasil (CAPES)—Finance Code 001 and also by Foundation for Research Support in Mato Grosso State—FAPEMAT (Fundação de Amparo a Pesquisa do Estado de Mato Grosso) Grant No. 366047/2017. Conselho Nacional de Desenvolvimento Científico e Tecnológico (National Council for Scientific and Technological Development), Grant Number 310181/2021-6 and Fundação Oswaldo Cruz (Oswaldo Cruz Foundation).

## Conflict of interest

The authors declare that the research was conducted in the absence of any commercial or financial relationships that could be construed as a potential conflict of interest.

## Publisher's note

All claims expressed in this article are solely those of the authors and do not necessarily represent those of their affiliated organizations, or those of the publisher, the editors and the reviewers. Any product that may be evaluated in this article, or claim that may be made by its manufacturer, is not guaranteed or endorsed by the publisher.
