# Supplementary material for: Genomic analysis of Mycobacterium tuberculosis variant bovis strains isolated from bovine in the state of Mato Grosso, Brazil
Source: Front Vet Sci. 2022 Nov 16;9:1006090. doi: 10.3389/fvets.2022.1006090 (PMC9709292; doi:10.3389/fvets.2022.1006090)
Supplement: Supplementary file 1 [file Data_Sheet_1.PDF]

Supplementary material 1 - List with identification samples SRA WGS

**BioSamplE**	**SRA**	**Country**	**Region**	**City**	**Reference**
SAMN15897433	SRR12511761	Brazil	São Paulo		Guimarães
SAMN16714398	SRR13015807	Brazil	Rio Grande do Sul	Gravatá	Lima et al., 2021
SAMN16714397	SRR13015808	Brazil	Rio Grande do Sul	Gravatá	Lima et al., 2021
SAMN16755322	SRR13046673	Brazil	Amazonas	Itacoatiara	Carneiro et al., 2021
SAMN16755329	SRR13046686	Brazil	Amazonas	Novo Céu	Carneiro et al., 2021
SAMN16755335	SRR13046680	Brazil	Pará	Prainha	Carneiro et al., 2021
SAMN21018177	SRR15649878	Brazil	Mato Grosso	Rondonópolis	Anjos et al., 2022
SAMEA5800050	ERR3445504	Brazil	Pará	Ilha de Marajó	Conceição et al., 2020
SAMEA5800032	ERR3445486	Brazil	Pará	Ilha de Marajó	Conceição et al., 2020
SAMEA5800037	ERR3445491	Brazil	Pará	Ilha de Marajó	Conceição et al., 2020
SAMEA5800038	ERR3445492	Brazil	Pará	Ilha de Marajó	Conceição et al., 2020
SAMEA5800039	ERR3445493	Brazil	Pará	Ilha de Marajó	Conceição et al., 2020
SAMEA5800041	ERR3445495	Brazil	Pará	Ilha de Marajó	Conceição et al., 2020
SAMEA5800042	ERR3445496	Brazil	Pará	Ilha de Marajó	Conceição et al., 2020
SAMEA5800043	ERR3445497	Brazil	Pará	Ilha de Marajó	Conceição et al., 2020
SAMEA5800044	ERR3445498	Brazil	Pará	Ilha de Marajó	Conceição et al., 2020
SAMEA5800046	ERR3445500	Brazil	Pará	Ilha de Marajó	Conceição et al., 2020
SAMEA5800034	ERR3445488	Brazil	Pará	Ilha de Marajó	Conceição et al., 2020
SAMEA5800045	ERR3445499	Brazil	Pará	Ilha de Marajó	Conceição et al., 2020
SAMEA7109736	ERR4450959	Brazil	Rio Grande do Sul	Ibirubá	Rodrigues et al., 2021
SAMEA7109707	ERR4450943	Brazil	Rio Grande do Sul	Eldorado do Sul	Rodrigues et al., 2021
SAMN08741066	SRR6865435	Brazil	São Paulo	São Paulo	Zimpel et al., 2020
SAMN08741066	SRR7693877	Brazil	São Paulo	São Paulo	Zimpel et al., 2020
SAMEA7109775	ERR4451187	Brazil	Rio Grande do Sul	Estrela	Rodrigues et al., 2021
SAMN16755339	SRR13046675	Brazil	Amazonas	Urucara	Carneiro et al., 2021
SAMEA7109693	ERR4450933	Brazil	Rio Grande do Sul	Teutônia	Rodrigues et al., 2021
SAMN16714403	SRR13015802	Brazil	Rio Grande do Sul	Gravatá	Lima et al., 2021
SAMN16714400	SRR13015805	Brazil	Rio Grande do Sul	Gravatá	Lima et al., 2021
SAMN16714395	SRR13015795	Brazil	Rio Grande do Sul	Gravatá	Lima et al., 2021
SAMN16714392	SRR13015798	Brazil	Rio Grande do Sul	Gravatá	Lima et al., 2021
SAMN21018176	SRR15649879	Brazil	Mato Grosso	Salto do Céu	Anjos et al., 2022
SAMN16714399	SRR13015806	Brazil	Rio Grande do Sul	Gravatá	Lima et al., 2021
SAMN12388335	SRR9850824	Brazil	São Paulo	São Paulo	Zimpel et al., 2020
SAMN16714390	SRR13015800	Brazil	Rio Grande do Sul	Gravatá	Lima et al., 2021
SAMN21018178	SRR15649877	Brazil	Mato Grosso	Lucas do Rio Verde	Anjos et al., 2022
SAMN16714391	SRR13015799	Brazil	Rio Grande do Sul	Gravatá	Lima et al., 2021
SAMEA5800047	ERR3445501	Brazil	Pará	Ilha de Marajó	Conceição et al., 2020
SAMN16714389	SRR13015801	Brazil	Rio Grande do Sul	Gravatá	Lima et al., 2021
SAMN16714402	SRR13015803	Brazil	Rio Grande do Sul	Gravatá	Lima et al., 2021
SAMN16714394	SRR13015796	Brazil	Rio Grande do Sul	Gravatá	Lima et al., 2021
SAMN12388336	SRR9850830	Brazil	São Paulo	São Paulo	Zimpel et al., 2020
SAMN16714396	SRR13015794	Brazil	Rio Grande do Sul	Gravatá	Lima et al., 2021
SAMN13961781	SRR10997360	Brazil			Sisco et al., 2020
SAMEA5800048	ERR3445502	Brazil	Pará	Ilha de Marajó	Conceição et al., 2020
SAMN16755325	SRR13046670	Brazil	Amazonas	Novo Céu	Carneiro et al., 2021
SAMN13961779	SRR10997362	Brazil			Sisco et al., 2020
SAMN16755324	SRR13046671	Brazil	Amazonas	Novo Céu	Carneiro et al., 2021
SRR13015804	SRR13015804	Brazil	Rio Grande do Sul	Gravatá	Lima et al., 2021
SAMN16755328	SRR13046687	Brazil	Amazonas	Novo Céu	Carneiro et al., 2021
SAMN16714393	SRR13015797	Brazil	Rio Grande do Sul	Gravatá	Lima et al., 2021
SAMN16755320	SRR13046677	Brazil	Amazonas	Careiro da Varzea	Carneiro et al., 2021
SAMEA5800031	ERR3445485	Brazil	Pará	Ilha de Marajó	Conceição et al., 2020
SAMN03288261	SRR6705904	Brazil	São Paulo		Guimarães et al., 2015
SAMEA5800033	ERR3445487	Brazil	Pará	Ilha de Marajó	Conceição et al., 2020

SAMN16755323	SRR13046672	Brazil	Amazonas	Manacapuru	Carneiro et al., 2021
SAMN16755327	SRR13046668	Brazil	Amazonas	Novo Céu	Carneiro et al., 2021
SAMN16755334	SRR13046681	Brazil	Amazonas	Parintins	Carneiro et al., 2021
SAMN16755338	SRR13046676	Brazil	Amazonas	Urucara	Carneiro et al., 2021
SAMN16714388	SRR13015809	Brazil	Rio Grande do Sul	Gravatá	Lima et al., 2021
SAMN13961780	SRR10997361	Brazil			Sisco et al., 2020
SAMEA7109771	ERR4451183	Brazil	Rio Grande do Sul	Arroio do Tigre	Rodrigues et al., 2021
SAMEA5800035	ERR3445489	Brazil	Pará	Ilha de Marajó	Conceição et al., 2020
SAMEA7109720	ERR4450950	Brazil	Rio Grande do Sul	Eldorado do Sul	Rodrigues et al., 2021
SAMEA6542014	ERR3906063	Brazil	Pará	Ilha de Marajó	Conceição et al., 2020
SAMEA7109780	ERR4451192	Brazil	Rio Grande do Sul	Camaquã	Rodrigues et al., 2021
SAMEA7109758	ERR4450970	Brazil	Rio Grande do Sul	Estrela	Rodrigues et al., 2021
SAMN16714387	SRR13015810	Brazil	Rio Grande do Sul	Gravatá	Lima et al., 2021
SAMN16755321	SRR13046674	Brazil	Amazonas	Itacoatiara	Carneiro et al., 2021
SAMN16755332	SRR13046683	Brazil	Amazonas	Novo Céu	Carneiro et al., 2021
SAMN16755331	SRR13046684	Brazil	Amazonas	Novo Céu	Carneiro et al., 2021
SAMN16755326	SRR13046669	Brazil	Amazonas	Novo Céu	Carneiro et al., 2021
SAMN16755337	SRR13046678	Brazil	Amazonas	Presidente Figueiredo	Carneiro et al., 2021
SAMN16755330	SRR13046685	Brazil	Amazonas	Novo Céu	Carneiro et al., 2021
SAMN16755336	SRR13046679	Brazil	Pará	Prainha	Carneiro et al., 2021
SAMN16755318	SRR13046689	Brazil	Amazonas	Apuí	Carneiro et al., 2021
SAMN21018175	SRR15649880	Brazil	Mato Grosso	Alta Floresta	Anjos et al., 2022
SAMEA5800049	ERR3445503	Brazil	Pará	Ilha de Marajó	Conceição et al., 2020
