# Supplementary material for: Genomic analysis of Mycobacterium tuberculosis variant bovis strains isolated from bovine in the state of Mato Grosso, Brazil
Source: Front Vet Sci. 2022 Nov 16;9:1006090. doi: 10.3389/fvets.2022.1006090 (PMC9709292; doi:10.3389/fvets.2022.1006090)
Supplement: Supplementary file 2 [file Data_Sheet_2.PDF]

Supplementary material 2 - Mutations of the strains

**SRR15649878 (TMT116)**	**SRR15649880 (TMT05)**	**SRR15649877 (TMT123)**	**SRR15649879(TMT24)**
Lineage Estimated Fraction Family Spoligotype Rd	Lineage Estimated Fraction Family Spoligotype Rd	Lineage Estimated Fraction Family Spoligotype Rd	Lineage Estimated Fraction Family Spoligotype Rd
lineageBOV 1.000 M. bovis BOV 1;BOV 2 None	lineageBOV 1.000 M. bovis BOV 1;BOV 2 None	lineageBOV 1.000 M. bovis BOV 1;BOV 2 None	lineageBOV 1.000 M. bovis BOV 1;BOV 2 None
lineageBOV AFRI 0.999	lineageBOV AFRI 1.000	lineageBOV AFRI 1.000	lineageBOV AFRI 1.000
Genome Position Locus Tag Gene Change Estimated Fraction			
2289073 Rv2043c pncA p.His57Asp 1.000	2289073 Rv2043c pncA p.His57Asp 1.000 pyrazinamide	2289073 Rv2043c pncA p.His57Asp 1.000 pyrazinamide	2289073 Rv2043c pncA p.His57Asp 1.000 pyrazinamide
5752 Rv0005 gyrB c.513G>A 1.000	5752 Rv0005 gyrB c.513G>A 0.996	5752 Rv0005 gyrB c.513G>A 1.000	5752 Rv0005 gyrB c.513G>A 1.000
6406 Rv0005 gyrB c.1167C>T 1.000	6406 Rv0005 gyrB c.1167C>T 1.000	6406 Rv0005 gyrB c.1167C>T 1.000	6406 Rv0005 gyrB c.1167C>T 1.000
6446 Rv0005 gyrB p.Ala403Ser 1.000	6446 Rv0005 gyrB p.Ala403Ser 1.000	6446 Rv0005 gyrB p.Ala403Ser 1.000	6446 Rv0005 gyrB p.Ala403Ser 1.000
7362 Rv0006 gyrA p.Glu21Gln 1.000	7362 Rv0006 gyrA p.Glu21Gln 1.000	7362 Rv0006 gyrA p.Glu21Gln 1.000	7362 Rv0006 gyrA p.Glu21Gln 1.000
7585 Rv0006 gyrA p.Ser95Thr 1.000	7585 Rv0006 gyrA p.Ser95Thr 1.000	7585 Rv0006 gyrA p.Ser95Thr 1.000	7585 Rv0006 gyrA p.Ser95Thr 1.000
8285 Rv0006 gyrA c.984C>T 1.000	7844 Rv0006 gyrA c.543C>T 1.000	8091 Rv0006 gyrA p.Arg264Cys 0.996	8091 Rv0006 gyrA p.Arg264Cys 1.000
9143 Rv0006 gyrA c.1842T>C 1.000	8285 Rv0006 gyrA c.984C>T 1.000	8285 Rv0006 gyrA c.984C>T 0.996	8285 Rv0006 gyrA c.984C>T 1.000
9217 Rv0006 gyrA p.Asp639Ala 1.000	9143 Rv0006 gyrA c.1842T>C 1.000	9143 Rv0006 gyrA c.1842T>C 1.000	9143 Rv0006 gyrA c.1842T>C 1.000
9304 Rv0006 gyrA p.Gly668Asp 1.000	9217 Rv0006 gyrA p.Asp639Ala 1.000	9217 Rv0006 gyrA p.Asp639Ala 1.000	9217 Rv0006 gyrA p.Asp639Ala 1.000
491742 Rv0407 fgd1 c.960T>C 1.000	9304 Rv0006 gyrA p.Gly668Asp 1.000	9304 Rv0006 gyrA p.Gly668Asp 1.000	9304 Rv0006 gyrA p.Gly668Asp 1.000
576737 Rv0486 mshA p.Val464Leu 1.000	491742 Rv0407 fgd1 c.960T>C 1.000	491742 Rv0407 fgd1 c.960T>C 1.000	491742 Rv0407 fgd1 c.960T>C 1.000
763031 Rv0667 rpoB c.3225T>C 1.000	763031 Rv0667 rpoB c.3225T>C 1.000	576108 Rv0486 mshA p.Ala254Gly 0.368	576108 Rv0486 mshA p.Ala254Gly 0.362
781395 Rv0682 rpsL c.-165T>C 1.000	781395 Rv0682 rpsL c.-165T>C 1.000	763031 Rv0667 rpoB c.3225T>C 1.000	763031 Rv0667 rpoB c.3225T>C 1.000
1302899 Rv1173 fbiC c.-32A>G 1.000	1302899 Rv1173 fbiC c.-32A>G 1.000	781395 Rv0682 rpsL c.-165T>C 1.000	781395 Rv0682 rpsL c.-165T>C 1.000
1417554 Rv1267c embR c.-207C>G 1.000	1417554 Rv1267c embR c.-207C>G 1.000	1302899 Rv1173 fbiC c.-32A>G 1.000	1302899 Rv1173 fbiC c.-32A>G 1.000
1834859 Rv1630 rpsA p.Ala440Thr 1.000	1834859 Rv1630 rpsA p.Ala440Thr 1.000	1417554 Rv1267c embR c.-207C>G 1.000	1417554 Rv1267c embR c.-207C>G 1.000

1917972 Rv1694 tlyA c.33A>G 1.000	1917972 Rv1694 tlyA c.33A>G 1.000	1476074 rrl rrl r.2417t>c 1.000	1476074 rrl rrl r.2417t>c 1.000
2154724 Rv1908c katG p.Arg463Leu 1.000	2154724 Rv1908c katG p.Arg463Leu 1.000	1834859 Rv1630 rpsA p.Ala440Thr 1.000	1834859 Rv1630 rpsA p.Ala440Thr 1.000
2155503 Rv1908c katG c.609G>A 1.000	2155503 Rv1908c katG c.609G>A 1.000	1917972 Rv1694 tlyA c.33A>G 1.000	1917972 Rv1694 tlyA c.33A>G 1.000
2156025 Rv1908c katG c.87G>T 1.000	2156025 Rv1908c katG c.87G>T 1.000	2154724 Rv1908c katG p.Arg463Leu 1.000	2154724 Rv1908c katG p.Arg463Leu 0.996
2518132 Rv2245 kasA c.18C>T 1.000	2518132 Rv2245 kasA c.18C>T 1.000	2155503 Rv1908c katG c.609G>A 1.000	2155503 Rv1908c katG c.609G>A 1.000
3086788 Rv2780 ald c.-32T>C 1.000	3086788 Rv2780 ald c.-32T>C 1.000	2156025 Rv1908c katG c.87G>T 1.000	2156025 Rv1908c katG c.87G>T 0.996
3087084 Rv2780 ald c.266 266del 1.000	3087084 Rv2780 ald c.266 266del 1.000	2518132 Rv2245 kasA c.18C>T 1.000	2518132 Rv2245 kasA c.18C>T 1.000
3642478 Rv3262 fbiB p.Asp315Ala 1.000	4240671 Rv3793 embC p.Thr270Ile 1.000	3086788 Rv2780 ald c.-32T>C 1.000	3086788 Rv2780 ald c.-32T>C 1.000
4240671 Rv3793 embC p.Thr270Ile 1.000	4242643 Rv3793 embC c.2781C>T 1.000	3087084 Rv2780 ald c.266 266del 1.000	3087084 Rv2780 ald c.266 266del 1.000
4242643 Rv3793 embC c.2781C>T 1.000	4242970 Rv3793 embC c.3108C>T 1.000	3642478 Rv3262 fbiB p.Asp315Ala 1.000	3642478 Rv3262 fbiB p.Asp315Ala 1.000
4242970 Rv3793 embC c.3108C>T 1.000	4244220 Rv3794 embA c.988C>T 1.000	4240671 Rv3793 embC p.Thr270Ile 1.000	4240671 Rv3793 embC p.Thr270Ile 1.000
4244220 Rv3794 embA c.988C>T 1.000	4246551 Rv3795 embB p.Asn13Ser 1.000	4242643 Rv3793 embC c.2781C>T 1.000	4242643 Rv3793 embC c.2781C>T 1.000
4246551 Rv3795 embB p.Asn13Ser 1.000	4246864 Rv3795 embB c.351C>T 1.000	4242970 Rv3793 embC c.3108C>T 1.000	4242970 Rv3793 embC c.3108C>T 1.000
4246864 Rv3795 embB c.351C>T 0.996	4247646 Rv3795 embB p.Glu378Ala 1.000	4244220 Rv3794 embA c.988C>T 1.000	4244220 Rv3794 embA c.988C>T 1.000
4247646 Rv3795 embB p.Glu378Ala 1.000	4269351 Rv3806c ubiA c.483G>A 1.000	4246551 Rv3795 embB p.Asn13Ser 1.000	4246551 Rv3795 embB p.Asn13Ser 1.000
4269351 Rv3806c ubiA c.483G>A 1.000	4269387 Rv3806c ubiA p.Glu149Asp 1.000	4246864 Rv3795 embB c.351C>T 1.000	4246864 Rv3795 embB c.351C>T 1.000
4269387 Rv3806c ubiA p.Glu149Asp 1.000	4269606 Rv3806c ubiA c.228A>G 1.000	4247646 Rv3795 embB p.Glu378Ala 1.000	4247646 Rv3795 embB p.Glu378Ala 1.000
4269606 Rv3806c ubiA c.228A>G 1.000	4407588 Rv3919c gid c.615T>C 1.000	4269351 Rv3806c ubiA c.483G>A 1.000	4269351 Rv3806c ubiA c.483G>A 1.000
4407588 Rv3919c gid c.615T>C 1.000		4269387 Rv3806c ubiA p.Glu149Asp 1.000	4269387 Rv3806c ubiA p.Glu149Asp 1.000
		4269606 Rv3806c ubiA c.228A>G 1.000	4269606 Rv3806c ubiA c.228A>G 1.000
		4407588 Rv3919c gid c.615T>C 1.000	4407588 Rv3919c gid c.615T>C 1.000
			4408199 Rv3919c gid p.Ser2Pro 1.000
