# Supplementary material for: Genomic analysis of Mycobacterium tuberculosis variant bovis strains isolated from bovine in the state of Mato Grosso, Brazil
Source: Front Vet Sci. 2022 Nov 16;9:1006090. doi: 10.3389/fvets.2022.1006090 (PMC9709292; doi:10.3389/fvets.2022.1006090)
Supplement: Supplementary file 3 [file Data_Sheet_3.PDF]

Supplementary material 3 - Drug resistance by tbprofiler

Strains	Genome Position	Locus	Tag Gene	Change	Estimated Fraction	Drug	Drug-resistance (tbprofiler)
SRR12511761	2289073	Rv2043c	pncA	p.His57Asp	1.000	pyrazinamide	Other
SRR13015807	2289073	Rv2043c	pncA	p.His57Asp	1.000	pyrazinamide	Other
SRR13015808	2289073	Rv2043c	pncA	p.His57Asp	1.000	pyrazinamide	Other
SRR13046673	2289073	Rv2043c	pncA	p.His57Asp	1.000	pyrazinamide	Other
SRR13046686	2289073	Rv2043c	pncA	p.His57Asp	1.000	pyrazinamide	Other
SRR13046680	2289073	Rv2043c	pncA	p.His57Asp	1.000	pyrazinamide	Other
SRR15649878 TMT116	2289073	Rv2043c	pncA	p.His57Asp	1.000	pyrazinamide	Drug-resistant
ERR3445504	6738	Rv0005	gyrB	p.Thr500Asn	1.000	ciprofloxacin, fluoroquinolones, levofloxacin, moxifloxacin, ofloxacin	Other
	1472307	rrs	rrs	r.462c>t	1.000	streptomycin	
ERR3445486	2289073	Rv2043c	pncA	p.His57Asp	1.000	pyrazinamide	Other
ERR3445491	2289073	Rv2043c	pncA	p.His57Asp	1.000	pyrazinamide	Other
ERR3445492	2289073	Rv2043c	pncA	p.His57Asp	0.993	pyrazinamide	Other
ERR3445493	2289073	Rv2043c	pncA	p.His57Asp	1.000	pyrazinamide	Other
ERR3445495	2289073	Rv2043c	pncA	p.His57Asp	1.000	pyrazinamide	Other
ERR3445496	2289073	Rv2043c	pncA	p.His57Asp	1.000	pyrazinamide	Other
ERR3445497	2289073	Rv2043c	pncA	p.His57Asp	0.992	pyrazinamide	Other
ERR3445498	2289073	Rv2043c	pncA	p.His57Asp	1.000	pyrazinamide	Other
ERR3445500	2289073	Rv2043c	pncA	p.His57Asp	0.994	pyrazinamide	Other
ERR3445488	2289073	Rv2043c	pncA	p.His57Asp	1.000	pyrazinamide	Other
ERR3445499	2289073	Rv2043c	pncA	p.His57Asp	1.000	pyrazinamide	Other
ERR4450959	2289073	Rv2043c	pncA	p.His57Asp	1.000	pyrazinamide	Other
ERR4450943	2289073	Rv2043c	pncA	p.His57Asp	1.000	pyrazinamide	Other
SRR6865435	2289073	Rv2043c	pncA	p.His57Asp	1.000	pyrazinamide	Other
SRR7693877	2289073	Rv2043c	pncA	p.His57Asp	1.000	pyrazinamide	Other
ERR4451187	2289073	Rv2043c	pncA	p.His57Asp	1.000	pyrazinamide	Other
SRR13046675	2289073	Rv2043c	pncA	p.His57Asp	1.000	pyrazinamide	Other
ERR4450933	2289073	Rv2043c	pncA	p.His57Asp	0.974	pyrazinamide	Other
SRR13015802	2289073	Rv2043c	pncA	p.His57Asp	1.000	pyrazinamide	Other
SRR13015805	2289073	Rv2043c	pncA	p.His57Asp	1.000	pyrazinamide	Other
SRR13015795	2289073	Rv2043c	pncA	p.His57Asp	1.000	pyrazinamide	Other
SRR13015798	2289073	Rv2043c	pncA	p.His57Asp	1.000	pyrazinamide	Other
SRR15649879 TMT24	2289073	Rv2043c	pncA	p.His57Asp	1.000	pyrazinamide	Drug-resistant
SRR13015806	2289073	Rv2043c	pncA	p.His57Asp	1.000	pyrazinamide	Other
SRR9850824	2289073	Rv2043c	pncA	p.His57Asp	1.000	pyrazinamide	Other
SRR13015800	2289073	Rv2043c	pncA	p.His57Asp	1.000	pyrazinamide	Other
SRR15649877 TMT123	2289073	Rv2043c	pncA	p.His57Asp	1.000	pyrazinamide	Drug-resistant
SRR13015799	2289073	Rv2043c	pncA	p.His57Asp	1.000	pyrazinamide	Other
	2289073	Rv2043c	pncA	p.His57Asp	1.000	pyrazinamide	
ERR3445501	2518476	Rv2245	kasA	p.Arg121Lys	0.478	isoniazid	Pre-MDR
	4247445	Rv3795	embB	p.Asp311Gly	0.483	ethambutol	
SRR13015801	2289073	Rv2043c	pncA	p.His57Asp	1.000	pyrazinamide	Other

SRR13015803	2289073	Rv2043c	pncA	p.His57Asp	1.000	pyrazinamide	Other
SRR13015796	2289073	Rv2043c	pncA	p.His57Asp	1.000	pyrazinamide	Other
SRR9850830	2289073	Rv2043c	pncA	p.His57Asp	1.000	pyrazinamide	Other
SRR13015794	2289073	Rv2043c	pncA	p.His57Asp	1.000	pyrazinamide	Other
	761110	Rv0667	rpoB	p.Asp435Val	1.000	rifampicin	
SRR10997360	2155168	Rv1908c	katG	p.Ser315Thr	1.000	isoniazid	MDR
	2289073	Rv2043c	pncA	p.His57Asp	1.000	pyrazinamide	
ERR3445502	2518476	Rv2245	kasA	p.Arg121Lys	0.900	isoniazid	Pre-MDR
SRR13046670	2289073	Rv2043c	pncA	p.His57Asp	1.000	pyrazinamide	Other
SRR10997362	761161	Rv0667	rpoB	p.Leu452Pro	0.993	rifampicin	Pre-MDR
	2289073	Rv2043c	pncA	p.His57Asp	0.985	pyrazinamide	
SRR13046671	2289073	Rv2043c	pncA	p.His57Asp	1.000	pyrazinamide	Other
SRR13015804	2289073	Rv2043c	pncA	p.His57Asp	1.000	pyrazinamide	Other
SRR13046687	2289073	Rv2043c	pncA	p.His57Asp	1.000	pyrazinamide	Other
SRR13015797	2289073	Rv2043c	pncA	p.His57Asp	1.000	pyrazinamide	Other
SRR13046677	2289073	Rv2043c	pncA	p.His57Asp	1.000	pyrazinamide	Other
ERR3445485	2289073	Rv2043c	pncA	p.His57Asp	1.000	pyrazinamide	Other
SRR6705904	2289073	Rv2043c	pncA	p.His57Asp	1.000	pyrazinamide	Other
ERR3445487	1472644	rrs	rrs	r.799c>t	0.683	streptomycin	Other
	2289073	Rv2043c	pncA	p.His57Asp	1.000	pyrazinamide	
SRR13046672	2289073	Rv2043c	pncA	p.His57Asp	1.000	pyrazinamide	Other
SRR13046668	2289073	Rv2043c	pncA	p.His57Asp	1.000	pyrazinamide	Other
SRR13046681	2289073	Rv2043c	pncA	p.His57Asp	1.000	pyrazinamide	Other
SRR13046676	2289073	Rv2043c	pncA	p.His57Asp	1.000	pyrazinamide	Other
SRR13015809	2289073	Rv2043c	pncA	p.His57Asp	1.000	pyrazinamide	Other
	761110	Rv0667	rpoB	p.Asp435Val	1.000	rifampicin	
SRR10997361	2155168	Rv1908c	katG	p.Ser315Thr	1.000	isoniazid	MDR
	2289073	Rv2043c	pncA	p.His57Asp	1.000	pyrazinamide	
ERR4451183	2289073	Rv2043c	pncA	p.His57Asp	1.000	pyrazinamide	Other
ERR3445489	2289073	Rv2043c	pncA	p.His57Asp	1.000	pyrazinamide	Other
ERR4450950	2289073	Rv2043c	pncA	p.His57Asp	1.000	pyrazinamide	Other
ERR3906063	2289073	Rv2043c	pncA	p.His57Asp	1.000	pyrazinamide	Other
ERR4451192	2289073	Rv2043c	pncA	p.His57Asp	1.000	pyrazinamide	Other
ERR4450970	2289073	Rv2043c	pncA	p.His57Asp	1.000	pyrazinamide	Other
SRR13015810	2289073	Rv2043c	pncA	p.His57Asp	1.000	pyrazinamide	Other
SRR13046674	2289073	Rv2043c	pncA	p.His57Asp	1.000	pyrazinamide	Other
SRR13046683	2289073	Rv2043c	pncA	p.His57Asp	1.000	pyrazinamide	Other
SRR13046684	2289073	Rv2043c	pncA	p.His57Asp	1.000	pyrazinamide	Other
SRR13046669	2289073	Rv2043c	pncA	p.His57Asp	1.000	pyrazinamide	Other
SRR13046678	2289073	Rv2043c	pncA	p.His57Asp	1.000	pyrazinamide	Other
SRR13046685	2289073	Rv2043c	pncA	p.His57Asp	1.000	pyrazinamide	Other
SRR13046679	2289073	Rv2043c	pncA	p.His57Asp	1.000	pyrazinamide	Other
SRR13046689	2289073	Rv2043c	pncA	p.His57Asp	1.000	pyrazinamide	Other
SRR15649880 TMT05	2289073	Rv2043c	pncA	p.His57Asp	1.000	pyrazinamide	Drug-resistant
	2289073	Rv2043c	pncA	p.His57Asp	1.000	pyrazinamide	
ERR3445503	2518476	Rv2245	kasA	p.Arg121Lys	0.577	isoniazid	Pre-MDR
	4247445	Rv3795	embB	p.Asp311Gly	0.375	ethambutol	
