# Supplementary material for: Genomic analysis of Mycobacterium tuberculosis variant bovis strains isolated from bovine in the state of Mato Grosso, Brazil
Source: Front Vet Sci. 2022 Nov 16;9:1006090. doi: 10.3389/fvets.2022.1006090 (PMC9709292; doi:10.3389/fvets.2022.1006090)
Supplement: Supplementary file 4 [file Data_Sheet_4.PDF]

Supplementary material 4 - RDscan_table

CHROM	START	END	LENGTH	LOF	RD	TYPE	Mbovis_AF212297	SRR15649877 - TMT123	SRR15649878 - TMT116	SRR15649879 - TMT24	SRR15649880 - TMT05
NC000962_3	104737	105012	239	Rv0095c		DEL	233				
NC000962_3	131931	132610	718	Rv0109		DEL					
NC000962_3	150020	150889	869	Rv0124	RD701	DEL					
NC000962_3	192244	192470	226	Rv0162c		DEL					
NC000962_3	264753	266655	1932	Rv0221,Rv0222,Rv0223c	RD10	DEL	1902	1902	1902	1902	1902
NC000962_3	308184	308456	272	Rv0256c		DEL					
NC000962_3	333671	338691	5020	Rv0278c,Rv0279c		DEL					
NC000962_3	340417	340642	225			DEL					
NC000962_3	361790	362910	1125	Rv0297		DEL					
NC000962_3	531388	531614	230	Rv0442c		DEL					
NC000962_3	623216	624285	1095	Rv0532		DEL					
NC000962_3	672000	675457	2286	Rv0578c		DEL			280		
NC000962_3	745282	745560	278	Rv0648		DEL					
NC000962_3	836192	840503	4384	Rv0747,Rv0746		DEL					
NC000962_3	887845	890729	2807	Rv0794c,Rv0795, Rv0796,Rv0797		DEL	2903	2890	417	2897	2887
NC000962_3	925345	927461	2348	Rv0833		DEL					
NC000962_3	964766	965172	282	Rv0867c		DEL	216				
NC000962_3	1053544	1053951	407	Rv0944,Rv0943c		DEL		407		407	
NC000962_3	1090778	1092004	1030	Rv0977		DEL					
NC000962_3	1169435	1169978	535			DEL					

NC000962_3	1188573	1191609	3028	Rv1067c,Rv1068c		DEL					
NC000962_3	1203261	1203578	317	Rv1078		DEL					
NC000962_3	1216946	1218533	1389	Rv1091		DEL					
NC000962_3	1385161	1386084	835	Rv1243c		DEL					
NC000962_3	1402932	1405936	3004	Rv1255c,Rv1257c,Rv1256c	RD13	DEL	3004	3004	3004	3004	3004
NC000962_3	1468104	1468919	815	Rv1313c		DEL					
NC000962_3	1488482	1489532	1069	Rv1325c		DEL					
NC000962_3	1523188	1547064	23876	Rv1360,Rv1365c,Rv1363c,Rv1368, Rv1370c,Rv1361c,Rv1359,Rv1373,Rv1362c,Rv1372, Rv1355c,Rv1358,Rv1366A,Rv1369c,Rv1357c,Rv1366, Rv1364c,Rv1371,Rv1356c,Rv1367c	RD145	DEL			23876		
NC000962_3	1533159	1533494	517	Rv1361c		DEL	263				335
NC000962_3	1541950	1543303	1353	Rv1369c,Rv1370c		DEL	1353	1353		1353	1353
NC000962_3	1572488	1572843	617	Rv1396c		DEL	356	356	356	356	356
NC000962_3	1618525	1618756	326			DEL					
NC000962_3	1630981	1634549	2569	Rv1450c		DEL					
NC000962_3	1633685	1634547	923			DEL		1009	965	208	1012
NC000962_3	1696015	1708747	12732	Rv1509,Rv1506c,Rv1511,Rv1507A,Rv1514c, Rv1516c,Rv1508A,Rv1507c,Rv1512,Rv1515c, Rv1508c,Rv1510,Rv1513	RD4	DEL	12732	12732	12732	12732	12732
NC000962_3	1768072	1768878	806	Rv1563c		DEL	806				
NC000962_3	1779277	1788505	9249	Rv1582c,Rv1575,Rv1578c,Rv1580c,Rv1574,		DEL					

				Rv1585c,Rv1573,Rv1581c,Rv1577c,Rv1586c, Rv1579c,Rv1584c,Rv1572c,Rv1583c,Rv1587c,Rv1576c							
NC000962_3	1846518	1847771	1253	Rv1638A,Rv1639c		DEL					
NC000962_3	1863890	1864259	369			DEL					
NC000962_3	1879368	1879659	287			DEL					
NC000962_3	1954608	1958781	4201	Rv1729c,Rv1728c,Rv1730c,Rv1731		DEL					
NC000962_3	1987701	1989056	2017	Rv1758,Rv1757c,Rv1756c		DEL	1355	1355		1355	1355
NC000962_3	1996099	1997463	1656	Rv1763,Rv1764		DEL	1386	1380		1387	1377
NC000962_3	2001205	2002319	1094	Rv1768		DEL					
NC000962_3	2025817	2028461	2644	Rv1790,Rv1789,Rv1788,Rv1787		DEL					
NC000962_3	2045670	2046319	761	Rv1803c	RD-sur1|RDoryx_1	DEL					1927
NC000962_3	2061420	2062129	672	Rv1818c		DEL					
NC000962_3	2088782	2089098	316	Rv1840c		DEL					
NC000962_3	2145786	2146095	286			DEL					
NC000962_3	2196710	2197271	561			DEL					
NC000962_3	2208004	2220723	12737	Rv1973,Rv1967,Rv1975,Rv1969,Rv1968,Rv1971, Rv1976c,Rv1977,Rv1970,Rv1974,Rv1966,Rv1964, Rv1972,Rv1965	RD7|RD713	DEL	12719	12719	12719	12719	12719
NC000962_3	2262710	2262927	216	Rv2015c		DEL					
NC000962_3	2305489	2306558	1048			DEL					
NC000962_3	2330072	2332100	2028	Rv2073c,Rv2074,Rv2075c,Rv2072c	RD9	DEL	2028	2028	2028	2028	2028

NC000962_3	2357137	2357880	505			DEL					
NC000962_3	2365413	2366767	1354	Rv2105,Rv2106		DEL	1354	1354		1354	1354
NC000962_3	2387364	2387699	302	Rv2126c		DEL					
NC000962_3	2393892	2394957	1065	Rv2134c,Rv2133c		DEL					
NC000962_3	2423527	2424280	747			DEL					
NC000962_3	2430115	2431470	1371	Rv2168c,Rv2167c		DEL	1355	1355		1355	1355
NC000962_3	2550012	2551365	1357	Rv2277c,Rv2279,Rv2278	RD182	DEL	1353	1353		1353	1353
NC000962_3	2626068	2636945	11134	Rv2349c,Rv2350c,Rv2352c,Rv2353c, Rv2348c,Rv2346c,Rv2355,Rv2351c,Rv2354,Rv2347c		DEL	10860	10860	10867	10860	10860
NC000962_3	2693070	2693580	483			DEL					
NC000962_3	2784615	2785968	1351	Rv2479c,Rv2480c		DEL	1353	1353		1353	1353
NC000962_3	2799820	2800361	541	Rv2488c		DEL					
NC000962_3	2801589	2805790	4081	Rv2490c		DEL					
NC000962_3	2864672	2864891	219			DEL					
NC000962_3	2881323	2882326	1003	Rv2562,Rv2561,Rv2563		DEL					
NC000962_3	2944265	2945052	1009	Rv2615c		DEL					
NC000962_3	2956749	2958714	2081	Rv2630,Rv2631,Rv2629		DEL					
NC000962_3	2970015	2980969	10992	Rv2647,Rv2656c,Rv2648,Rv2657c,Rv2652c, Rv2653c,Rv2659c,Rv2646,Rv2651c,Rv2649, Rv2650c,Rv2658c,Rv2645,Rv2654c,Rv2655c		DEL	10954	10954	10954	10954	10954
NC000962_3	2969338	2986226	16888	Rv2647,Rv2656c,Rv2663,Rv2666,Rv2648,Rv2657c, Rv2662,Rv2651c,Rv2667,Rv2670c,Rv2660c,Rv2654c,		DEL					

				Rv2655c,Rv2668,Rv2661c,Rv2665, Rv2669,Rv2652c,Rv2653c,Rv2659c, Rv2646,Rv2649,Rv2650c,Rv2664,Rv2658c,Rv2645							
NC000962_3	3119221	3119775	943			DEL			1209		
NC000962_3	3147081	3147356	347	Rv2839c		DEL					
NC000962_3	3244502	3245476	974	Rv2931,Rv2930		DEL					
NC000962_3	3382072	3382605	1012			DEL					
NC000962_3	3426604	3427989	1385	Rv3063,Rv3062		DEL					
NC000962_3	3481465	3482003	536		RDcan|RD12oryx	DEL					
NC000962_3	3485073	3487513	2534	Rv3118,Rv3119,Rv3121,Rv3120	RDcan|RD12oryx|RD12	DEL	2515	2443	2476	2448	2463
NC000962_3	3551228	3554066	2838	Rv3187,Rv3186,Rv3185,Rv3184	RDoryx_4	DEL	2838	2838		2838	2838
NC000962_3	3710380	3711735	1355	Rv3326,Rv3325		DEL	1355	1355	1355	1355	1355
NC000962_3	3732727	3736228	3401			DEL	3199	2933	2932	2920	5243
NC000962_3	3732729	3742252	9511	Rv3344c,Rv3343c,Rv3345c		DEL					
NC000962_3	3741960	3742377	587			DEL					
NC000962_3	3766789	3767022	343		RDRio	DEL					
NC000962_3	3779508	3779889	373			DEL					
NC000962_3	3795056	3796411	1355	Rv3380c,Rv3381c		DEL	1355	1355		1355	1355
NC000962_3	3802155	3803826	1578	Rv3388		DEL					
NC000962_3	3817365	3824973	7608	Rv3403c,Rv3402c,Rv3401, Rv3400,Rv3405c,Rv3404c		DEL					

NC000962_3	3842621	3847485	4891	Rv3429,Rv3426,Rv3427c,Rv3425, Rv3428c		DEL	4826	4842	4842	4842	4841
NC000962_3	3884192	3884807	615	Rv3467	ND2	DEL					
NC000962_3	3890775	3892134	1359	Rv3474,Rv3475		DEL	1359	1359	1359	1359	1359
NC000962_3	3897073	3897784	711			DEL	711	711	711	711	711
NC000962_3	3927126	3936337	9402	Rv3508,Rv3507		DEL					
NC000962_3	3932967	3934690	1352	Rv3508		DEL					266
NC000962_3	3943645	3944485	899			DEL		849			
NC000962_3	3940067	3950071	9925	Rv3511,Rv3512,Rv3513c,Rv3514		DEL			9770		
NC000962_3	3946345	3949749	3453	Rv3514		DEL		3370		3395	3374
NC000962_3	4031791	4032594	887	Rv3590c		DEL					
NC000962_3	4036905	4037282	625	Rv3595c		DEL					
NC000962_3	4053162	4053400	291	Rv3611		DEL					
NC000962_3	4056839	4062732	5930	Rv3622c,Rv3623,Rv3617,Rv3620c,Rv3618,Rv3619c,Rv3621c	RD8|RD236a	DEL	5893	5893	5893	5893	5893
NC000962_3	4174793	4175448	655	Rv3728		DEL					
NC000962_3	4189592	4190759	1167	Rv3739c,Rv3738c	N-RD25das|N-RD25_tbB|N-RD25_tbA|N-RD25bov/cap	DEL	1167	1167	1167	1167	1167
NC000962_3	4222221	4222423	202	Rv3776		DEL					
NC000962_3	4253576	4254345	770	Rv3798		DEL		770	775	775	774

NC000962_3	4304843	4305880	1031	Rv3830c,Rv3831,Rv3829c		DEL					
NC000962_3	4309962	4310217	255			DEL					
NC000962_3	4353185	4353886	732	Rv3876	RD1mic|RD1bcg|RD1das	DEL					
NC000962_3	4350264	4361120	10848	Rv3880c,Rv3871,Rv3873,Rv3872, Rv3875,Rv3881c,Rv3879c,Rv3878,Rv3874,Rv3877,Rv3876	RD1mic|RD1bcg|RD1das	DEL					
NC000962_3	4370877	4374372	3199	Rv3887c,Rv3891c,Rv3890c,Rv3889c,Rv3888c	RD252|RDbovis	DEL	3351	3351	3351	3351	3351
NC000962_3	4389007	4390731	1724	Rv3903c,Rv3904c		DEL					
